# Impact of the transition to HPV-based primary screening in Portugal's organized cervical cancer screening program: A controlled interrupted time-series analysis (2014-2023)

**DOI:** 10.1016/j.puhip.2026.100792

**Published:** 2026-04-24

**Authors:** Rita Sousa, José Alberto Fonseca-Moutinho, Fábio Gomes, Fernanda Loureiro, Ana Rita Goes, Patrícia Soares

**Affiliations:** a NOVA National School of Public Health, Public Health Research Centre, Comprehensive Health Research Center, CHRC, REAL, CCAL, NOVA University Lisbon, Av. Padre Cruz, 1600-560, Lisbon, Portugal; b Portuguese Institute of Oncology of Coimbra, Francisco Gentil, Av. Bissaya Barreto 98, 3000-075, Coimbra, Portugal; c Health Sciences School, University of Beira Interior, Rua Marquês D'Ávila e Bolama, 6201-001, Covilhã, Portugal; d Public Health Department, Central Region Health Administration, Av. Dom Afonso Henriques, 141, 3001-553, Coimbra, Portugal; e National Institute of Health, Doutor Ricardo Jorge, Av. Padre Cruz, 11, 1649-016, Lisboa, Portugal

**Keywords:** Early detection of cancer, Uterine cervical neoplasms, Mass screening, HPV DNA tests, Interrupted time series analysis, Health policy evaluation

## Abstract

**Objectives:**

To evaluate the impact of transitioning from cytology to primary human papillomavirus (HPV) testing on cervical cancer screening (CCS) performance in Portugal's Central Region.

**Study design:**

Retrospective, population-based evaluation using a controlled interrupted time-series (CITS) approach.

**Methods:**

CCS registry data (2014–2023) were analyzed in six-month intervals. Three performance indicators (participation, test positivity, and priority referrals) were modeled using negative binomial regression with appropriate offsets. A COVID-19 covariable (national lockdown, March–June 2020) adjusted for the temporary suspension of screening services. The organized breast cancer screening (BCS) program served as an external control to distinguish CCS-specific effects from system-wide temporal fluctuations.

**Results:**

The analysis included 594,074 CCS and 888,184 BCS screening episodes. Following HPV implementation, CCS evolved differently from the control group across all outcomes. Priority referrals showed the strongest effect, with a four-fold immediate increase in CCS not observed in BCS (IRR: 4.31; 95% CI: 3.89-4.79). Participation and test positivity also diverged between programs, although with smaller magnitude changes. Post-intervention trends differed across all outcomes, although the COVID-19 pandemic, occurring shortly after implementation, complicates the interpretation of temporal patterns.

**Conclusions:**

Transition to HPV-based screening was associated with changes in screening processes, including increased identification of high-risk cases requiring priority referral while maintaining participation. By incorporating an external control, the CITS approach strengthens attribution of observed effects to HPV implementation rather than background system dynamics. These findings support HPV-based screening within organized programs and highlight its role in improving risk stratification and program performance.

## 1 Introduction

Cervical cancer (CC) remains a major public health challenge, ranking as the fourth most common cancer among women worldwide. In 2022, an estimated 660,000 new cases and 350,000 deaths were reported, with the highest burden in low- and middle-income countries [1]. To accelerate global elimination, the World Health Organization (WHO) established the “90–70–90” targets: 90% HPV vaccination coverage, 70% screening with a high-performance test by ages 35 and 45, and 90% access to timely treatment for precancer and cancer by 2030 [2].

Organized screening programs have substantially reduced CC incidence and mortality in settings with high population coverage and quality assurance [3,4]. Historically, cytology-based Pap smears formed the cornerstone of cervical cancer screening (CCS). However, extensive evidence shows that primary HPV testing has superior sensitivity for detecting high-grade precursor lesions [[5], [6], [7], [8], [9]]. As a result, international guidelines now recommend HPV-based primary screening [2,3,10], and many countries have transitioned to this approach [11].

Experiences from early adopters show heterogeneous effects on screening performance following HPV rollout, including participation and test positivity, depending on program maturity and implementation approach. In some settings, participation temporarily declined due to extended screening intervals or organizational changes, whereas in others, diagnostic yield and risk stratification improved substantially [8,[12], [13], [14], [15], [16]]. These variations underscore the need for population-based evaluations to ensure that transition to HPV testing strengthens both program effectiveness and equity [14].

In Portugal, the organized CCS program has progressively expanded nationwide since the 1990s [17]. The Central Region was the first to implement an organized, cytology-based program [18] and subsequently transitioned to primary HPV testing in 2019 [19]. Other regions implemented organized screening later, with some adopting HPV testing from inception. This makes the Central Region a unique setting to assess real-world effects of transitioning from cytology to HPV testing. Despite this pioneering role, few studies have evaluated the long-term impact of this transition on core performance indicators, including participation, test positivity, and referral indicators.

Evaluating the real-world impact of major screening reforms requires analytical approaches capable of distinguishing intervention effects from concurrent external shocks that may influence screening performance over time. The COVID-19 pandemic constituted an unprecedented system-wide disruption to preventive health services. In Portugal, organized screening activities were suspended or substantially reduced during lockdown periods, leading to sharp declines in screening participation and cancer detection [19]. At the Portuguese Oncology Institute of Porto, CC diagnoses fell by 74.3% between March and July 2020 compared with 2019, with a higher proportion of cases presenting at advanced stages [20]. Similar disruptions were reported internationally, raising concerns about delayed diagnosis and potential increases in advanced disease [[21], [22], [23]].

Robust quasi-experimental methods are therefore required to distinguish the impact of planned programmatic changes, such as the transition to HPV screening, from unplanned system-wide disruptions [24]. Interrupted time-series (ITS) analysis, particularly when incorporating an external control, is among the strongest approaches for evaluating population-level interventions while accounting for concurrent confounding [[25], [26], [27]]. This methodology has been widely applied to assess screening policy changes [28,29], pandemic-related service disruptions [23,30], and quality improvement initiatives [31].

### 1.1 Aims of this study

This study evaluates the effect of transitioning from cytology-to HPV-based primary screening in Portugal's Central Region on three performance indicators: participation, test positivity, and priority referrals. The Central Region's mature screening infrastructure provides an opportunity to assess program performance while accounting for concurrent disruptions from the COVID-19 pandemic.

## 2 Methods

### 2.1 Study design and setting

We conducted a retrospective population-based evaluation of the organized CCS program in Portugal's Central Region from January 2014 to December 2023. A controlled ITS (CITS) design was used to estimate population-level changes associated with the transition from cytology-based to primary HPV screening while accounting for temporal trends.

The organized CCS program targets women aged 25–64 years and operates through a call–recall system coordinated at the regional level by the Regional Health Administrations (RHA) and implemented through primary care units. During the cytology-based phase, women aged 25–64 years were invited every three years [17]. Following the transition to HPV-based screening, the target age range was adjusted to 25–60 years with a five-year screening interval [19]; however, women aged 60–64 years remained temporarily eligible as they completed screening rounds initiated under the previous protocol.

The transition to HPV testing occurred gradually between March and September 2019 and was treated as a transition phase, with the intervention point defined as September 2019 (full implementation).

The organized breast cancer screening (BCS) program, targeting women aged 50–69 years, follows a biennial invitation schedule based on a regionally coordinated call–recall system [17,19]. Program coordination, including identification of the eligible population and monitoring, is ensured by RHA. However, screening activities (invitations and mammography) are implemented by external entities, primarily the Portuguese League Against Cancer (LPCC), often using mobile units. Although delivery differs, overall coordination and population coverage are consistent across programs, supporting the comparability of temporal trends.

Both programs are integrated within the public health system, rely on centralized registries for population identification, invitation, and follow-up, and are monitored through standardized national indicators.

BCS was used as an external negative control, as it shares key organizational features with CCS (population-based approach, call–recall system, and regional coordination) but did not undergo protocol changes during the study period, allowing distinction between CCS-specific effects and broader system-level trends.

Differences in CCS screening periodicity resulted in varying proportions of the target population being invited per semester. In addition, between 2019 and 2022, the overlap of cytology- and HPV-based invitation cycles temporarily affected the denominators of eligible women and should be considered when interpreting participation trends.

All indicators were aggregated into six-month intervals (January–June, July–December) to ensure stable and comparable estimates over time.

A more detailed description of both screening programs is provided in Supplementary Table S1.

### 2.2 Data sources

Data were extracted from (1) *SiiMA Rastreios* [32], the regional registry containing invitations, participation, test type and result, cytology triage, and referral priority for CCS and BCS; and (2) *SIARS* (Public Health Department registries) [33], providing denominators for eligible and target populations by program, semester, and health unit.

Population estimates were obtained from Statistics Portugal [34]. All data were pseudonymized before analysis. The Regional Health Administration authorized data access and processing in accordance with national data protection regulations.

### 2.3 Selection of participants

We included aggregated data for all women eligible for the organized CCS program according to national age-based criteria between January 2014 and December 2023. Records covered all screening episodes performed within the organized program, using either cytology or HPV testing, depending on the implementation phase.

The BCS program contributed to the external control series. Because no protocol changes occurred in BCS during the study period, it allowed adjustment for system-wide temporal effects (e.g., service disruptions, health-system fluctuations) unrelated to HPV implementation.

### 2.4 Screening test classification and referral

Cytology results were classified according to the Bethesda system [35], with abnormal cytology defined as atypical squamous cells of undetermined significance (ASC-US) or worse. HPV tests were categorized as negative, HPV 16/18 positive, or other high-risk HPV (hrHPV) positive with reflex cytology, following national guidelines [36].

Priority referrals included cases with high-grade cytology (ASC–H, AGC, HSIL, AIS, SCC, ADC), HPV 16/18 positivity, or hrHPV positivity with high-grade reflex cytology. Non-priority referrals included cases with low-grade cytology (ASC–US/LSIL) or hrHPV positivity with low-grade reflex cytology. This classification aligns with risk-based management principles from the 2019 ASCCP consensus guidelines, which stratify immediate colposcopy risk based on HPV genotype and cytology severity [37].

### 2.5 Exposure

The exposure of interest was the transition to primary HPV testing, defined as fully implemented from September 2019 onwards. The pre-intervention period was January 2014–August 2019 (cytology-based screening), and the post-intervention period was September 2019–December 2023 (HPV-based screening).

### 2.6 Outcome measures

Screening performance was evaluated using three indicators (participation rate, test positivity rate, and priority referral rate) selected in accordance with international quality frameworks for organized CCS [3,[38], [39], [40], [41]]. These indicators reflect the core dimensions recommended for monitoring program reach, test performance, and diagnostic workload in HPV-based systems.

Participation rate, defined as the proportion of eligible women screened per semester, is the primary measure of program reach and equity and is consistently recommended in the European Guidelines and WHO/IARC (International Agency for Research on Cancer) monitoring frameworks [3,39].

Test positivity rate, defined as the proportion of positive results among valid screening tests, is a standard indicator of screening test performance and, in HPV-based systems, reflects analytical sensitivity and changes in underlying HPV prevalence or assay performance [3,41,42].

Priority referral rate, defined as the proportion of screened women referred for urgent assessment due to high-risk findings, corresponds to the “referral for assessment” indicators included in European quality assurance documents [3,38]. Risk-stratified referral is an important part of contemporary HPV-based screening systems (Netherlands, Denmark, Australia), as well as the American Society for Colposcopy and Cervical Pathology (ASCCP) 2019 risk-based management approach, providing a meaningful indicator of triage performance and downstream diagnostic demand in HPV programs [3,37,39,40].

All indicators were calculated for six-month intervals. Definitions and calculation methods are summarized in Table 1. Descriptive data on screen-detected and unscreened ICC were obtained for contextual interpretation but were not included in the ITS models since we did not have access to a longer time series (Supplementary Table S2).

**Table 1:** **–** Indicator definitions, data sources, and calculations for both cervical cancer and breast cancer screening programs.

Indicator	Definition	Data Source(s)	Calculation^a^
**Participation Rate** [38,50]	% of eligible women screened per period	SIARS, SiiMA	screened/eligible per period x 100
**Positive test rate** [38,50]	% of positive screening tests among all tests	SiiMA	positive/screened x 100
**Proportion of Priorities** [38,50]	% of women prioritized for referral among all tests	SiiMA	priorities/screened x 100

a All indicators were assessed at six-month intervals.

### 2.7 Statistical analysis

Descriptive statistics (absolute and relative frequencies, medians, and first and third quartiles Q1-Q3) summarized trends across the study period.

To estimate the effect of the transition to HPV-based screening, we fitted negative binomial generalized linear models to account for overdispersion. Time (in semesters) modeled pre-intervention trend. Two parameters captured the intervention effect: 1) level change, representing the immediate shift at full HPV implementation (September 2019); and 2) slope (time-after) change, capturing differences in the post-intervention trend.

Offsets were defined according to each outcome: the eligible population for participation models and the number of screened women for test positivity and priority referral models.

A CITS specification was applied by incorporating the BCS series as an external comparator. Program-by-period interaction terms (Level × BCS; Time-after × BCS) were included to estimate CCS-specific effects relative to the BCS counterfactual and separate them from system-wide temporal patterns.

Pandemic-related disruption was adjusted for using a COVID-19 variable (0 = pre-pandemic period, 1 = suspension period (national lockdown, March-June 2020), and 2 = recovery/normalization period (July-December and thereafter). This covariable captured the pre-pandemic baseline, the suspension of screening during lockdown, and the subsequent recovery period.

Model results were expressed as incidence rate ratios (IRRs) with 95% confidence intervals (CIs) and interpreted as percentage changes relative to baseline trends. Model diagnostics included visual inspection of residuals and assessment of autocorrelation and partial autocorrelation (ACF/PACF). All analyses were performed in R 4.2.3 for CITS modeling and IBM SPSS Statistics version 29.0.1.0 for descriptive statistics.

## 3 Results

**Fig. 1:**
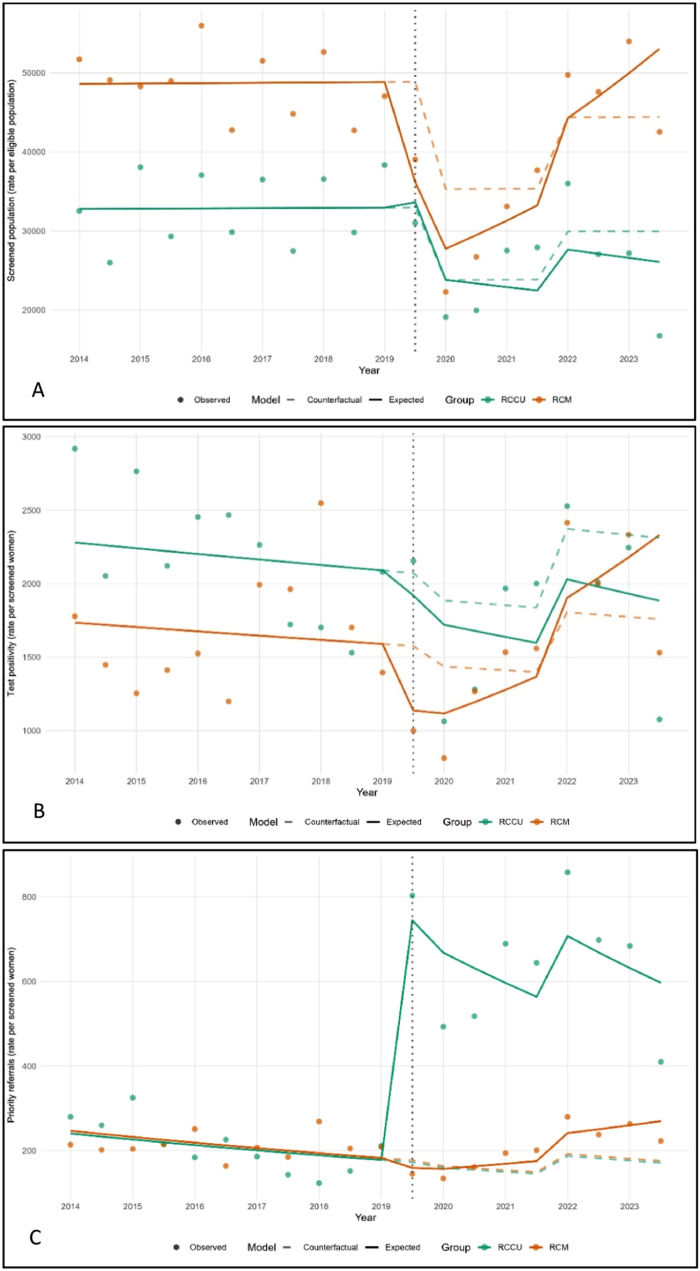
**CITS plots for comparing cervical (CCS, green) and breast (BCS, orange) cancer screening programs in Portugal's Central Region (2014**–**2023)**. A) participation rate, B) test positivity rate; C), priority referral rate. Solid lines represent observed values, and dashed lines represent modeled counterfactuals (expected trends without intervention). Vertical dotted line marks HPV implementation (September 2019).

The analysis included all eligible women in Portugal's Central Region between 2014 and 2023 (target population approximately 1.63 million). Over this period, 594,074 CCS and 888,184 BCS episodes were recorded. Semester-level CCS volumes declined from a median of 32,546 under cytology to 27,193 after HPV implementation, while participation increased slightly (41.9% to 45.0%) (Table 2). This divergence reflects the longer 5-year HPV interval and overlapping invitation cycles between 2019 and 2022. Test positivity remained stable (6.7% cytology vs. 7.0% HPV), whereas priority referrals increased more than fourfold (0.56% vs. 2.52%) after HPV implementation (Table 2, Fig. 1C).

**Table 2:** Screening indicators before and after transition to HPV-based screening.

Screening indicators	Cytology-based period Median (Q1-Q3)	HPV-based period Median (Q1-Q3)	Total study period Median (Q1-Q3)
**Screened population**			
• **CCS** a	32,546 (29,320–37,069)	27,193 (19,528–29,468)	29,565 (27,085–36,387)
• **BCS**	48,957 (29,912–48,668)	39,051 (29,912–48,668)	47,324 (39,919–51,071)

**Proportion of screened (%)**			
• **CCS**	41.9 (39.4-51.2)	45.0 (34.7-51.3)	44.1 (38.9-51.2)
• **BCS**	64.5 (61.0-73.2)	63.4 (48.7-78.2)	64.3 (59.9-75.4)

**Positive test rate (%)**			
• **CCS**	6.7 (5.5-8.1)	7.0 (6.4-7.3)	7.0 (6.3-7.4)
• **BCS**	3.0 (2.8-4.0)	4.2 (3.6-4.7)	3.8 (2.9-4.4)

**Priority referrals (%)**			
• **CCS**	0.56 (0.52-0.87)	2.52 (2.42-2.59)	0.95 (0.5-2.5)
• **BCS**	0.42 (0.41-0.45)	0.53 (0.50-0.60)	0.47 (0.41-0.53)

Q1–Q3 = first and third quartiles; CCS – cervical cancer screening; BCS: breast cancer screening.

a Note: Eligibility denominators reflect the screening interval (3-year cytology: 1/6 of target cohort per semester vs. 5-year HPV: 1/10). During 2019–2022, overlapping rounds produced temporary discrepancies.

No seasonality pattern was detected.

CITS models showed that CCS evolved differently from BCS following HPV implementation (Table 3). Participation rates evolved differently in CCS compared to BCS after HPV implementation (Fig. 1A). The immediate change differed by 27% between programs (IRR:0.73; 95%CI: 0.67-0.79), and post-intervention trends diverged significantly, with CCS increasing 8% per semester relative to BCS (IRR:1.08; 95%CI: 1.07-1.10). The COVID-19 lockdown resulted in a 28% reduction in CCS participation (IRR:0.72; 95%CI: 0.66-0.79). We found a similar pattern for test positivity. The immediate change differed by 22% between CCS and BCS (IRR:0.78; 95%CI: 0.68-0.89), and post-intervention trends diverged significantly, with CCS increasing 10% per semester relative to BCS (IRR:1.10; 95%CI: 1.07-1.13) (Fig. 1B). COVID-19 lockdown and normalization periods did not significantly affect test positivity in CCS.

**Table 3:** Effect of HPV transition on participation, test positivity, and priority referrals, adjusted for pandemic-related disruptions: results from CITS analysis.

Parameter	IRR∗	95% CI∗	*p*-value
Participation rate			
Time (trend)	1.00	1.00 - 1.01	0.900
Level (HPV)	1.02	0.94 - 1.11	0.600
Time-after (HPV slope)	0.98	0.96 - 1.00	0.066
COVID-19 covariate			
• **Lockdown**	0.72	**0.66**–**0.79**	**<0.001**
• Covid-19 normalization	0.91	0.79 - 1.04	0.200
**Control Group (BCS)**	**1.48**	**1.43**–**1.54**	**<0.001**
**Level x BCS**	**0.73**	**0.67**–**0.79**	**<0.001**
**Time-after x BCS**	**1.08**	**1.07**–**1.10**	**<0.001**
**Test positivity**			
Time (trend)	0.99	0.98 - 1.00	0.059
Level (HPV)	0.93	0.81 - 1.06	0.300
Time-after (HPV slope)	0.98	0.95 - 1.02	0.400
COVID-19 covariate			
• Lockdown	0.92	0.80 - 1.05	0.200
• Covid-19 normalization	1.20	0.95 - 1.50	0.120
**Control Group (BCS)**	**0.76**	**0.72**–**0.81**	**<0.001**
**Level x BCS**	**0.78**	**0.68**–**0.89**	**<0.001**
**Time-after x BCS**	**1.10**	**1.07**–**1.13**	**<0.001**
**Priority referrals**			
**Time(trend)**	**0.97**	**0.96**–**0.98**	**<0.001**
**Level (HPV)**	**4.31**	**3.89**–**4.79**	**<0.001**
Time-after (HPV slope)	0.97	0.95 - 1.00	0.055
COVID-19 covariate			
• Lockdown	0.95	0.85 - 1.06	0.300
• **Covid-19 normalization**	**1.26**	**1.05**–**1.51**	**0.011**
Control Group (BCS)	1.03	0.98 - 1.08	0.300
**Level x BCS**	**0.21**	**0.19**–**0.23**	**<0.001**
**Time-after x BCS**	**1.10**	**1.08**–**1.12**	**<0.001**

IRR = Incidence Rate Ratio, CI = Confidence Interval; BCS: breast cancer screening.

Bold values indicate statistically significance at p < 0.05; COVID-19 lockdown refers to the national suspension of screening activities (March-June 2020).

Priority referrals displayed the strongest HPV-specific effect. CCS diverged dramatically from BCS: the immediate change differed by 79% between programs (IRR:0.21; 95%CI: 0.19-0.23), reflecting a substantial four-fold increase in CCS (IRR:4.31; 95%CI: 3.89-4.79). Post-intervention trends also diverged, with CCS increasing 10% per semester compared to BCS (IRR:1.10; 95%CI: 1.08-1.12) (Fig. 1C). The COVID-19 lockdown did not significantly affect priority referrals in CCS (IRR:0.95; p = 0.30), though the normalization period was associated with a 26% increase (IRR:1.26; 95%CI: 1.05-1.51).

Descriptive cancer outcomes (Supplementary Table S2) showed that screen-detected CC increased from 0.24 to 0.44 per 1000 women screened between the cytology and HPV periods, while CC incidence among unscreened women remained stable (7.45 vs. 7.00 per 100,000).

## 4 Discussion

This CITS study examined the transition from cytology to HPV-based primary screening in Portugal's Central Region over a ten-year period. By comparing CCS with the BCS program as an external counterfactual, it was possible to distinguish HPV-related effects from background system-wide trends. The most notable finding was a four-fold increase in priority referrals in CCS, not observed in BCS, consistent with increased identification of high-risk cases. Participation and test positivity rates also evolved differently between programs, although with smaller magnitude changes.

Participation patterns diverged between CCS and BCS after HPV implementation, with differences in immediate changes and post-intervention trends. Despite these shifts, participation remained stable. Differences in participation between programs are expected due to variations in target populations, screening intervals, and perceived disease risk, which may limit comparability. Nevertheless, BCS provides a useful approximation of system-wide trends affecting both programs. Similar stability in participation has been reported in Sweden, Finland, and Italy, where established invitation systems helped maintain engagement despite extended screening intervals [12,[43], [44], [45]].

Test positivity also followed a distinct trajectory in CCS compared to BCS, with significant immediate differences and diverging trends, while remaining stable in absolute terms within CCS. This pattern is consistent with early implementation phases in other HPV-based programs, where positivity may shift modestly due to assay characteristics, testing algorithms, and transient pools of previously undetected infections. Evidence from the Netherlands, Sweden, Finland, and Australia suggests that HPV-based screening may alter positivity patterns without large immediate increases, particularly in settings with long-standing cytology-based screening [5,14,15,43,45].

Priority referrals showed the clearest HPV-specific effect, with both immediate and sustained increases relative to the control series. This pattern is consistent with the expected impact of HPV-based screening on risk stratification and downstream diagnostic demand. Similar increases in high-grade referrals, colposcopy use, or detection of cervical intraepithelial neoplasia grade 2 or worse (CIN2+) have been reported following HPV implementation in several countries [5,7,15,[45], [46], [47]], typically stabilizing after the first screening round, as prevalent CIN2+ lesions are detected and treated [12]. Complementary descriptive data from this study, showing higher screen-detected CC with stable incidence among unscreened women, are consistent with patterns reported in previous studies following HPV implementation [5,8,9]. However, small numbers preclude formal modeling, and no conclusions can be drawn regarding clinical effectiveness or diagnostic accuracy.

The temporary decline in participation during the COVID-19 lockdown reflects the national suspension of preventive services rather than program-specific effects. Parallel reductions observed in BCS support the interpretation that these changes were driven by system-wide disruptions, consistent with reports from multiple European countries [21,48], the United States [22], and Japan [23], where screening activity dropped sharply in early 2020 and progressively recovered thereafter.

### 4.1 Strengths and limitations

This study has many strengths. The CITS design strengthens causal inference by comparing CCS changes with a contemporaneous external comparator exposed to the same health-system pressures. Population-level data coverage, multiple performance indicators, and an extended pre- and post-intervention period provide a robust assessment of screening performance during a major programmatic transition.

Limitations include structural differences between CCS and BCS, including target age, screening periodicity, underlying disease epidemiology, and differences in operational delivery, which may introduce residual confounding despite shared organizational frameworks. The overlap of 3- and 5-year invitation cycles between 2019 and 2022 may have affected participation denominators, complicating short-term interpretation. Registry data are subject to delays and inconsistencies inherent to routine information systems. Increased priority referrals may partly reflect differences between cytology- and HPV-based triage pathways, and linkage to histopathology and staging data will be necessary to determine whether these changes represent true gains in diagnostic efficiency. In addition, the study does not include histologically confirmed outcomes (e.g., CIN2+), preventing direct evaluation of diagnostic accuracy, false positives, or clinical effectiveness. Finally, interpretation of post-intervention trends is complicated by the COVID-19 pandemic, which occurred shortly after HPV implementation. Although we adjusted for pandemic effects and used BCS as a control, differential impacts between programs cannot be fully excluded.

Overall, HPV-based primary screening was associated with changes in screening processes, including increased identification of high-risk cases requiring referral, while maintaining stable participation within the expected ranges for mature programs. These findings support the implementation of HPV testing within organized screening systems and highlight the importance of ensuring adequate diagnostic capacity during early transition phases. Methodologically, this study reinforces the value of CITS approaches for evaluating screening reforms and other health system interventions, particularly in the presence of concurrent external disruptions. Future research should incorporate histopathological outcomes, biomarker-based triage, cost-effectiveness analyses, and stratified evaluations by age, socioeconomic status and vaccination status, to strengthen long-term monitoring of program performance and equity.

### 4.2 Conclusion

Using a CITS design, this study distinguished the effects of Portugal's transition to HPV-based primary screening from broader system-wide trends. Comparison with an external control showed that the transition was associated with strengthened risk stratification and increased referral of high-risk cases, while maintaining participation.

The temporary decline observed during the COVID-19 lockdown reflected a national suspension of preventive services and did not bias HPV-related estimates. Overall, these findings support HPV-based screening within organized programs and underscore the importance of continuous performance monitoring and adequate diagnostic capacity during the transition period.

## Ethical statement

Ethical approval for this study was obtained from the Ethics Committee of the Central Region Health Administration and from Ethics Committee of the Portuguese Institute of Oncology of Coimbra, Francisco Gentil. The study was conducted in accordance with the ethical standards of the institutional and national research committees and with the Declaration of Helsinki and its later amendments [49].

## Authors’ contributions

All authors contributed substantially to the conceptualisation, writing (review and editing) and approved the final manuscript for submission. R.S. ARG, J.A.F.M. and P.S. designed the methodology, validated the data, and oversaw project administration; R.S., P.S. conducted the formal analysis and drafted the manuscript. ARG and PS are joint last authors and contributed equally to this work.

## Funding

This work was partially supported by the 10.13039/501100019370Foundation for Science and Technology (reference: CEECINST/00049/2021/CP2817/CT0001 and https://doi.org/10.54499/CEECINST/00049/2021/CP2817/CT0001).

## Declaration of competing interest

The authors declare that they have no known competing financial interests or personal relationships that could have appeared to influence the work reported in this paper.
