# Supplementary material for: Impact of the transition to HPV-based primary screening in Portugal's organized cervical cancer screening program: A controlled interrupted time-series analysis (2014-2023)
Source: Public Health Pract (Oxf). 2026 Apr 24;11:100792. doi: 10.1016/j.puhip.2026.100792 (PMC13157080; doi:10.1016/j.puhip.2026.100792)
Supplement: Multimedia component 1 [file mmc1.docx]

**Appendix A. Supplementary data**

**Supplementary Table S1: key characteristics of organized screening programs in Portugal**

| **Characteristic** | **Cervical Cancer Screening (CCS)** | **Breast Cancer Screening (BCS)** |
| --- | --- | --- |
| Target population | Women aged 25–64 years (later 25–60) | Women aged 50–69 years |
| Screening test | Cytology / HPV DNA test | Mammography |
| Screening interval | 3 years / 5 years | 2 years |
| Implementation | Primary care units | External providers (mainly LPCC) |
| Coordination | Regional Health Administrations (RHA) | Regional Health Administrations (RHA) |
| Invitation system | Call/recall via primary care  (invitation by letter, telephone, or in-person contact) | Call/recall via external providers (invitation by letter) |
| Screening delivery | Primary care settings  (fixed units) | Mobile and fixed screening units |
| Transition period | March–September 2019 | No protocol change |
| Data system | Centralized registries | Centralized registries |
| Role in study | Intervention group | External negative control |

LPCC – Portuguese League Against Cancer

**Organizational model of CCS and BCS in Portugal**

**Cervical cancer screening (CCS):**

1. RHA identify the eligible population
2. Invitation via primary care units
3. Screening test performed in primary care
4. Centralized laboratory processing
5. Referral to colposcopy units for diagnosis, treatment, and follow-up

**Breast cancer screening (BCS):**

1. RHA identify the eligible population
2. Invitation managed by external entities (mainly LPCC)
3. Screening performed via mobile and fixed units
4. Image reading and referral
5. Referral to hospital-based breast units for diagnosis, treatment, and follow-up

**Supplementary Table S2 :** Invasive cervical cancer (ICC) detection before and after transition to HPV-based screening in Portugal’s Central Region, 2014–2023

| **Outcome** | **Cytology-based period (<=2019)** | **HPV-based period (2020-2023)** | **Relative change (%)** |
| --- | --- | --- | --- |
| **Screen-detected ICC (per 1,000 screened)** | 0.24 | 0.44 | **+83%** |
| **ICC among unscreened women (per 100,000)** | 7.45 | 7.00 | **–6%** |

**Notes:**Data are descriptive and were not included in interrupted time-series (ITS) models due to small annual case counts.

Estimates are based on regional registry linkage between SiiMA Rastreios and hospital morbidity data (GDH), aggregated by screening period.

ICC: invasive cervical cancer; HPV: human papillomavirus***.***
